# Suspected community-acquired pneumonia in an ambulatory setting (CAPA): a French prospective observational cohort study in general practice

**DOI:** 10.1038/npjpcrm.2015.10

**Published:** 2015-03-12

**Authors:** Henri Partouche, Céline Buffel du Vaure, Virginie Personne, Chloé Le Cossec, Camille Garcin, Alain Lorenzo, Christian Ghasarossian, Paul Landais, Laurent Toubiana, Serge Gilberg

**Affiliations:** 1 Département de médecine générale, Faculté de médecine, Sorbonne Paris Cité, Université Paris Descartes, Paris, France; 2 Service d’informatique médicale et de biostatistique, Hôpital Necker Enfants Malades, Paris, France; 3 Collège National des Généralistes Enseignants (CNGE), France; 4 Equipe d’accueil 24-15, Institut Universitaire de Recherche Clinique, Université Montpellier 1, Montpellier, France; 5 Inserm Umrs 1142 LIMICS, Centre de Recherche des Cordeliers, Université Pierre et Marie Curie, UPMC, Paris, France

## Abstract

**Background:**

Few studies have addressed the pragmatic management of ambulatory patients with suspected community-acquired pneumonia (CAP) using a precise description of the disease with or without chest X-ray (X-ray) evidence.

**Aims:**

To describe the characteristics, clinical findings, additional investigations and disease progression in patients with suspected CAP managed by French General Practitioners (GPs).

**Methods:**

The patients included were older than 18 years, with signs or symptoms suggestive of CAP associated with recent-onset unilateral crackles on auscultation or a new opacity on X-ray. They were followed for up to 6 weeks. Descriptive analyses of all patients and according to their management with X-rays were carried out.

**Results:**

From September 2011 to July 2012, 886 patients have been consulted by 267 GPs. Among them, 278 (31%) were older than 65 years and 337 (38%) were at increased risk for invasive pneumococcal disease. At presentation, the three most common symptoms, cough (94%), fever (93%), and weakness or myalgia (81%), were all observed in 70% of patients. Unilateral crackles were observed in 77% of patients. Among patients with positive radiography (64%), 36% had no unilateral crackles. A null CRB-65 score was obtained in 62% of patients. Most patients (94%) initially received antibiotics and experienced uncomplicated disease progression regardless of their management with X-rays. Finally, 7% of patients were hospitalised and 0.3% died.

**Conclusions:**

Most patients consulting GPs for suspected CAP had the three following most common symptoms: cough, fever, and weakness or myalgia. More than a third of them were at increased risk for invasive pneumococcal disease. With or without X-rays, most patients received antibiotics and experienced uncomplicated disease progression.

## Introduction

Early diagnosis of community-acquired pneumonia (CAP) is essential for prompt initiation of an empirical antimicrobial therapy as stated in guidelines to improve patient outcomes.^1–3^

However, the diagnosis of CAP can be complex, as it is often based on a combination of clinical symptoms, radiographic, laboratory and microbiological findings, and the clinical response to antimicrobial therapy. Previous reviews have shown that patient history and physical examination are not sufficient to reliably differentiate CAP from other lower respiratory tract infections.^4–6^

Consequently, findings suggestive of pneumonia on chest X-ray (X-ray) are considered a gold standard and are recommended in order to confirm the diagnosis.^1–3^

In primary care, patients frequently consult in the early stage of the disease and the clinical findings classically described are thus seldom all present. An X-ray is not always taken for patients, nor is it a part of the General Practitioner's (GP's) decision-making process.^7^ Moreover, the optimal usefulness of an X-ray in the assessment of suspected CAP is not consensual between all guidelines.^1–3,8,9^

To our knowledge, no large studies taking into account the pragmatic management of patients with suspected CAP in ambulatory practice have primarily focussed on a precise description of the disease. Furthermore, most ambulatory studies have focussed on symptoms and signs of confirmed CAP using X-rays in patients consulting their GP for cough or lower respiratory tract infection symptoms and not in patients with suspected CAP.^10–13^

The primary objective of this study was to describe patients with clinical findings suggestive of CAP and managed in general practice in terms of past history, presenting manifestations, clinical findings, radiological and/or biological results and disease progression (recovery, hospitalisation or death). The secondary objective was to describe patients according to their management with or without X-rays.

## Materials and methods

This prospective cohort study was conducted between 21 September 2011 and 2 July 2012. A network of 475 French GPs had to include consecutively all adult outpatients with suspected CAP. Data were collected in an electronic case report form (eCRF).

### Study population

All investigators were part of a national GP network involved in clinical research. They were recruited through a website linked to the eCRF. A stratified random sampling based on a multistage geographical cluster at a departmental level was used.

Patient inclusion criteria were as follows: age older than 18 years and having recently experienced one or more signs suggestive of acute pneumonia, such as fever >38.5 °C, cough, chest pain, tachycardia >100 beats/min, tachypnoea >25 breaths/min, and clinical global impression of severity. These criteria had to be associated with one focus of recent-onset unilateral crackles on auscultation. In the absence of unilateral crackles, a new opacity on X-ray was mandatory to definitely include the patient. For this purpose, a pre-inclusion process was possible in the eCRF until a positive X-ray was obtained. Then, GPs could register patient characteristics and clinical findings recorded at the initial visit.

Patients hospitalised in the previous month were not included. Patients who fulfilled the inclusion criteria had to sign an informed consent form before data collection during the baseline visit. As patients were followed in the context of current patient management practices, investigations and treatments were at the GP's initiative. The number of follow-up visits was not pre-specified. Only the final visit (by consultation or telephone contact) was mandatory and had to be done between the fourth and sixth week following inclusion (regardless of whether the patient had recovered).

### Data collection

The collected data included visit date, age, gender, lifestyle, history and clinical findings, investigations and their results, treatments, reasons for reconsulting, duration of main symptoms, sick leave and its duration if present, hospitalisation and death. Patient data were de-identified.

We considered patients at increased risk for invasive pneumococcal disease as those with chronic respiratory disease (COPD, emphysema, chronic bronchitis, chronic pulmonary failure), asthma, chronic heart failure, nephrotic syndrome, aspleny or splenectomy, homozygous sickle cell disease, HIV infection, diabetes mellitus or previous history of pneumonia.^14,15^ Severity was assessed using the five-point CRB-65 score including confusion, respiratory rate ⩾30/min, systolic blood pressure <90 mm Hg or diastolic blood pressure ⩽60 mm Hg and age ⩾65 years.^16^

A positive radiograph was defined by the presence of focal alveolar opacity or multiple, mottled, peribronchial opacities or localised or diffuse interstitial opacities. A negative radiograph was defined by normal or nonspecific radiographic findings and/or isolated pleural effusion.^4,8^

Two clinical research associates performed a data quality control process during the whole study period. They focussed their control on record completeness and data collection from additional investigations. The monitoring team ensured that inclusion criteria were met, particularly in patients without X-ray or with negative radiography to rule out bronchitis or crackles due to other causes.

A periodical online newsletter was sent to the investigators to avoid patients lost to follow-up.

### Data analysis

Continuous data are presented as mean (standard deviation) for parametric distributions, or as median [interquartile range] for non-parametric distributions. Categorical data are presented as frequencies (percentages). Percentages were calculated on data available; the percentage of unavailable data (%NA) is provided for each variable in the tables. Percentages were compared using Pearson’s Chi-square test and means using Student’s *t*-test. All the tests were two-sided and performed with a 5% type-I error. The analysis was performed using R 3.0.2 Software.^17^

### Study sample size

Assuming that patients with X-rays should represent 60% of the sample^7^ and that 10% of patient records could be incomplete, a sample of 1,056 patients consisting of 634 patients with X-rays were therefore necessary to have a power of 90% leading to a precision of 5% in descriptive statistics with a type-I error of 5%.

## Results

**Figure 1:**
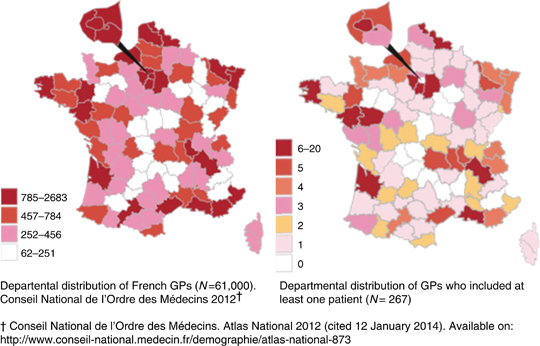
Departmental distribution of investigators who participated in the CAPA study. CAPA, community-acquired pneumonia in an ambulatory setting; GP, general practitioner.

**Figure 2:**
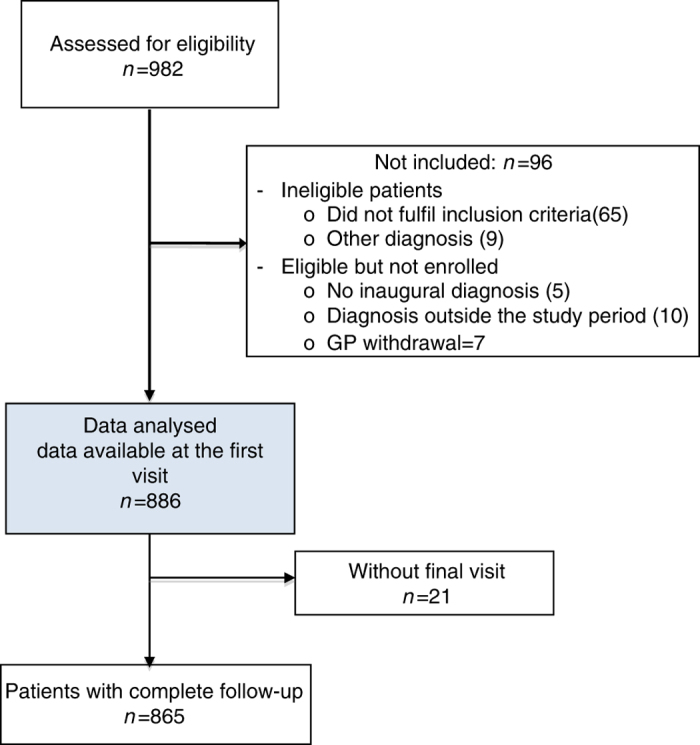
Flowchart of patients included. GP, general practitioner.

Within the national training network, 425 selected investigators accepted to participate and 267 (63%) included at least one patient (Figure 1, Table 1). Between 21 September 2011 and 2 July 2012, 886 patients with suspected CAP were included. Data collected at the final visit were available for 865 patients (Figure 2).

### Characteristics and past history

The median age of the patients was 55 (18–102) years and 278 (31%) were older than 65. Among the patients included, 314 (35%) were retired, 43 (5%) received free health care (low income), 27 (3%) were unemployed or disabled and 25 (3%) were students. Four hundred and five (46%) patients reported no medical history, whereas 337 (38%) patients were at increased risk for invasive pneumococcal disease. Other main results are presented in Table 2. In addition, 58 (7%) were diabetic, 28 (3%) were treated with long-term steroids or immunosuppressive agents and 53 (6%) with long-term psychotropic drugs. Two hundred and three (23%) patients had received an influenza vaccine for the season 2011–2012 and 94 (11%) a 23-valent pneumococcal polysaccharide vaccine.

### Symptoms and physical signs at inclusion

Sudden onset of symptoms was reported by 62% of patients and the median time before consulting was 4 (2–7) days. The three most common symptoms at presentation were cough, temperature >37.8 °C and weakness or myalgia (Table 2). Among the 798 patients with data available, 558 (70%) experienced all three of the most common symptoms.

On chest auscultation, 95 (11%) patients had normal breath sounds and 709 (80%) had crackles, including 684 (77%) with unilateral crackles and 273 (31%) with unilateral isolated crackles. Among patients with crackles, 245 (28%) had rhonchi and 150 (17%) had wheezing.

A clinical global impression of severity was reported in 240 (27%) patients. When calculating the Gennis’ prediction rule,^18^ 744 (92%) patients had at least one altered vital sign (bottom of Table 2).

Confusion and hypotension (systolic blood pressure <90 mm Hg or diastolic blood pressure ⩽60 mm Hg) were observed in 16 (2%) and 8 (1%) patients, respectively. Among the 642 patients with data available, 399 (62%) patients obtained a null score on the CRB-65.^16^ Other results are presented in Table 2.

### Additional medical investigations

One hundred and seventy-two (19%) patients were managed without X-rays. Among the 714 patients with X-rays, 233 (33%) underwent it within 3 days following their inclusion visit and the remaining patients after 3 days. The median time to obtain a positive and negative radiographic result was 5 (3–9) and 6 (4–11) days, respectively. Finally, 563 (64%) patients presented a positive radiograph, including 202 (36%) patients without unilateral crackles. Among the 558 patients who had all three most common symptoms, 447 (80%) had undergone a chest X-ray, which was positive in 356 (80%).

White blood cell count was taken in 316 (36%) patients; C-reactive protein (CRP) level was assessed in 314 (35%) patients; and procalcitonin level was evaluated in 13 (1%) patients. *Legionella* and *Streptococcus pneumoniae* urinary antigens were searched in 2 and 0% of patients, respectively.

### Treatments prescribed at the inclusion visit

Most patients (94%) were prescribed an antibiotic at the inclusion visit. A first-line monotherapy with amoxicillin, co-amoxi-clavulanic acid, macrolide and pristinamycin was prescribed to 357 (43%), 251(30%), 88 (10%) and 49 (6%) patients, respectively. The median dose and duration of amoxicillin treatment were 3 g/day for 10 days. Among the patients not treated with amoxicillin or co-amoxi-clavulanic acid, 35 (23%) were allergic to penicillin. A first-line bitherapy was prescribed for 34 (4%) patients and included a macrolide in 21 (2%) patients.

### Follow-up, evolution and death

Five hundred and seventeen (58%) and 120 (13%) patients had one and two follow-up visits, respectively. The median duration between the inclusion visit and the first follow-up visit was 7 (3–11) days. Among the patients who had a first follow-up visit, 83 (16%) had a change in their antimicrobial therapy that included 20 (4%) additions of a second antibiotic and 64 (12%) antibiotic switches, including 13 (3%) switches from amoxicillin or co-amoxi-clavulanic acid to a macrolide.

Finally, 62 (7%) patients were hospitalised and 3 (0.3%) died during the follow-up period. Patients at increased risk for invasive pneumococcal disease and patients with CRB-65 score ⩾1 were more often hospitalised than others (respectively, 10 vs. 5%, *P*=0.002, and 13 vs. 3%, *P*<0.001). Other results are presented in Table 3.

### Description of subgroups according to the use of chest radiography

Patients older than 65 years were more often managed without having undergone an X-ray (25 vs. 17% for patients younger than 65, *P*<0.003). Among patients managed with X-rays, those older than 65 years had a negative chest radiography more frequently compared with patients younger than 65 years (26 vs.19%; *P*=0.04). Other results related to patient characteristics at the inclusion visit and disease progression are presented in Table 2 and Table 3, respectively.

## Discussion

### Main findings

In this cohort of 886 patients with suspected CAP, 31% were older than 65 years and 38% were at increased risk for invasive pneumococcal disease. At presentation, most patients had cough, fever, and weakness or myalgia, and 70% of them experienced all three of these most common symptoms. Most patients (92%) had at least one vital sign altered, 77% had unilateral crackles and 11% had no abnormality on chest auscultation. Among the 563 (64%) patients who had positive radiography, one third had no unilateral crackles. Two thirds of patients with data available obtained a null CRB-65 score. Regardless of their management with X-rays, most patients received antibiotics and most experienced an uncomplicated disease progression,

### Interpretation of findings in relation to previously published work

#### Clinical findings

In our population, cough and fever, two of the three most common symptoms observed, were very frequent, as previously reported in other studies that also included patients with suspected CAP.^7,19^ Concomitantly, we found that sputum production, dyspnoea and unilateral chest pain, which are common clinical features of confirmed CAP, were less frequently present, but these results were in accordance with those of a previous study.^20^ The proportion of patients with unilateral crackles on chest auscultation was high (77%) but comparable to the result of a study with similar design.^7^ When considering only patients with positive radiography, the proportion of patients with unilateral crackles remained high (64%) compared with those reported in previous studies conducted on patients with lower respiratory tract infection: 20%^11^ and up to 32%^10^ in patients with new pulmonary infiltrate. This could be due to our inclusion criteria that favoured the selection of patients with unilateral crackles on chest auscultation. It is likely that most GPs could not determine whether the crackles were recent or chronic. However, these results confirm that, although crackles are an essential diagnostic criterion, they have limited value in predicting a pulmonary infiltrate when they are used alone.^6^

#### Contribution of chest radiography and co-morbidity

Our investigators were allowed to pragmatically manage their patients in accordance with the European guidelines in which an X-ray is not systematically included in the management of patients with suspected CAP.^8,9^ However, using an X-ray remains the cornerstone of the diagnosis according to French guidelines, which include an infiltrate on X-ray in the definition of CAP.^1,2^ It is probably the reason why GPs, among whom 89% were medical trainers, used X-rays in most patients (80%) and probably not in selected patients with uncertain diagnosis or progressive disease or at risk for underlying lung pathology.^9^

Dyspnoea, described elsewhere as a predictor of pulmonary infiltrate in patients with lower respiratory tract infection,^10,21^ was less frequently observed in patients with positive radiography than in patients with negative radiography (44 vs. 56%, *P*=0.04; Table 2). Similar results were observed for unilateral chest pain and for sputum or phlegm (33 vs. 47%, *P*<0.01, and 51 vs. 68%, *P*<0.01).

Our inclusion critera could have led GPs to include more patients with dyspnoea, unilateral chest pain and sputum among patients with negative radiography as patients with atypical symptoms (without crackles) had to have a mandatory positive radiography to be included. However, results for sputum are consistent with those of Hopstaken *et al.*, who have identified dry cough as an independent and significant predictor of pulmonary infiltrate.^22^ Wheezing, a common symptom of COPD known to interfere with the clinical features of pneumonia,^23^ was also less frequently observed in patients with positive radiography (17 vs. 30%, *P*<0.01). Similarly, there were fewer patients older than 65 years and with chronic respiratory disease among those with positive radiography than among those with negative radiography (respectively, 27 vs. 36%, *P*=0.02, and 10 vs. 15%, *P*<0.01). However, there were close but low proportions of patients without ‘vital signs’ of the Gennis’ score^18^ among those with either positive or negative radiography or without X-rays (Table 2). These results suggest that X-rays have limited value in ruling out pneumonia,^24^ especially in patients with chronic respiratory disease and in elderly patients.

#### Antibiotic treatments

A systematic treatment with antibiotics was observed in our population, regardless of the prognostic factors. In fact, 69% of patients were younger than 65 years, 46% had no former medical history, and 62% of patients with data available obtained a null CRB-65 score. The median dose and duration of the most prescribed antibiotic (amoxicillin, 1 g three times daily for 10 days) were higher and longer than those recommended by the UK Guidelines^9^ (amoxicillin, 500 mg three times daily for 7 days). Most GPs were probably convinced to treat patients with CAP and strictly met the French Guidelines, which emphasise the importance of initiating high-dose amoxicillin within 4 h after diagnosis.^1,2^ However, this strategy, which is not recommended in several European countries, remains questionable as long-term treatment could be linked to the emergence and selection of resistant bacteria^25^ and this dosage is associated with a higher incidence of gastritis and diarrhoea.^26^

#### Disease progression

Most patients had mild disease with uncomplicated progression during the follow-up period, regardless of their management with X-rays. We can assume that patients presenting with severe disease could have consulted at the emergency departments. However, our proportions of hospitalisation and death were consistent with those reported in a previous population-based study.^27^ In addition, our results confirm the importance of identifying patients at increased risk for invasive pneumococcal disease^14,15^ or with high CRB-65 score^16^ as these patients were more often hospitalised in our population.

### Strengths and limitations of this study

The main strengths of our study were its design and sample size. Our inclusion criteria were close to those of the current practice as they allowed GPs to include patients without any context of cough. Investigators were evenly distributed throughout France. Compared with the national medical demography,^28,29^ the rates of men, medical trainers, rural location or group exercise were larger in our group of investigators (Table 1). The patients included mainly lived in a rural or semi-urban environment and their socio-professional distribution was similar to the French demographic distribution.^30^

However, our study has some limitations. First, the investigators were part of a trainer and research GP network. Thus, the representativeness of the GP practice could be questioned. However, it has been shown that results of practice-based research networks are relevant to other practising clinicians.^31^ Second, according to our inclusion criteria, an X-ray was not performed in all patients. GP decision-making process, care conditions, patient preferences and other causes could have influenced the decision to perform or not perform an X-ray.^32^ We did not ask the investigators the reason for prescribing or not prescribing an X-ray as our objectives were focussed on patient characterisation.

Furthermore, when an X-ray was performed, the interpretation by a local radiologist aware of the suspected diagnosis could have contributed to overdiagnosis, particularly in case of non-alveolar pneumonia.^33^ However, as our study took place in the ambulatory setting, forcing a strict timing to perform the CR associated with a double reading was not consistent with the current practice.

In patients with negative radiography but with clinical findings consistent with CAP, false negatives with delayed positivity were possible. However, the median time for obtaining negative radiography results following the inclusion was 6 days and only 33 patients had a negative X-ray within the 3 days following the inclusion. Moreover, obtaining a normal X-ray despite pulmonary infection confirmed microbiologically or by chest CT has been previously described.^34^

### Implications for future research, policy and practice

Our findings support the questionable usefulness of the routine X-ray in the management of patients with suspected CAP.^35^ Further ambulatory studies with microbiological investigations and/or biological markers are needed to help GPs in their decision to prescribe antibiotics.

### Conclusions

At first presentation, most patients consulting GPs for suspected CAP had a sudden onset of cough, fever, and weakness or myalgia. All of these three most common symptoms were found in 70% of patients. Most patients had mild disease even if a significant proportion of them were at increased risk for invasive pneumococcal disease. However, systematic antibiotic prescription, with mainly high-dose amoxicillin, was observed in this population. Regardless of their management with X-rays, most patients experienced an uncomplicated disease progression. These findings confirm the low relevance of a routine X-ray in assessing suspected CAP in primary care.

## Figures and Tables

**Table 1:** Characteristics of GPs who included at least one patient (active), GPs who did not (non-active) and the National demography of French GPs

	*Active GPs (*n*=267)* n *(%)*	*Non-active GPs (*n*=158)* n *(%)*	P *value*	*National demography* a *(%)*
*Gender*				
Women	80 (30.0)	39 (24.7)	NS	(41.2)
				
*Age*				
30–39 years	54 (20.2)	26 (16.4)		(17.7)
40–49 years	67 (25.1)	34 (21.5)		(28.1)
50–59 years	105 (39.3)	76 (48.1)	NS	(37.6)
Older than 60 years	41 (15.4)	22 (13.9)		(16.6)
				
*Location*				
Rural <5,000 inhabitants^b^	80 (30.0)	52 (32.9)		(12.1)
Semi-rural	71 (26.6)	31 (19.6)	NS	(16.6)
Urban >20,000	116 (43.4)	75 (47.5)		(71.3)
				
Group practice	191 (71.5)	31 (19.6)	<0.001	(45.3)
Teaching physician	238 (89.1)	136 (86.1)	NS	(9.5)
No alternative medicine^c^	253 (94.8)	155 (98.1)	NS	(90.1)

Abbreviations: GP, general practitioner; NS, not significant.

a Sicart D. Direction de la recherche, des études, de l’évaluation et des statistiques (DREES). Ministère du travail, de l'emploi et de la santé. Les médecins au 1er janvier 2012. Available on: http://www.drees.sante.gouv.fr/les-professions-de-sante-au-1er,10930.html. Accessed 14 July 2014.

b Living in a city of <5,000 inhabitants.

c No partial activity of acupuncture or homoeopathy or osteopathy.

**Table 2:** Characteristics, symptoms, signs at inclusion of all patients and patients with positive, negative and without chest radiographs

	*All included patients*, N*=886*		*No chest radiography*, N*=172*		*Positive chest radiography*, N*=563*		*Negative chest radiography,* N*=151*		P *value*
	n *(%)* a *or mean [s.d.]*	*NA* b *(%)*	n *(%)* a *or mean [s.d.]*	*NA* b *(%)*	n *(%)* a *or mean [s.d.]*	*NA* b *(%)*	n *(%)* a *or mean [s.d.]*	*NA* b *(%)*	
Gender, female	467 (53)	—	91 (53)	—	299 (53)	—	77 (51)	—	0.89
Age ⩾65 years	278 (31)	—	70 (41)	—	154 (27)	—	54 (36)	—	<0.01
BMI (kg/m^2^)	25 [5]	(5.3)	25 [5]	(5.8)	25 [5]	(4.8)	26 [5]	(6.3)	<0.01
Location: rural <5,000 inhab^c^	366 (41)	—	77 (45)	—	227 (40)	—	62 (41)	—	0.67
Children younger than 5 years at home	182 (21)	(0.9)	24 (14)	(1.2)	130 (23)	(0.5)	28 (19)	(2.0)	0.23
Home-dependent patients	46 (5)	—	14 (8)	—	25 (4)	(0.2)	7 (5)	—	0.15
Previous 23-valent PPV	94 (11)	—	19 (11)	—	59 (10)	—	16 (11)	—	0.41
Smoker	205 (23)	(0.5)	49 (28)	—	116 (21)	(0.7)	40 (26)	—	0.12
Previous CAP	124 (14)	—	29 (17)	—	82 (15)	—	13 (9)	—	0.08
Asthma	100 (11)	—	19 (11)	—	56 (10)	—	25 (17)	—	0.07
Chronic respiratory disease	118 (13)	—	38 (22)	—	57 (10)	—	23 (15)	—	<0.01
Heart failure	42 (5)	—	11 (6)	—	22 (4)	—	9 (6)	—	0.30
Cough	833 (94)	—	163 (95)	—	523 (93)	—	147 (97)	—	0.10
Weakness or myalgia	718 (81)	—	137 (80)	—	465 (83)	—	116 (77)	—	0.24
Chills	616 (70)	(0.1)	115 (67)	—	399 (71)	(0.2)	102 (68)	—	0.49
Sputum or phlegm	505 (57)	(0.1)	115 (67)	—	288 (51)	(0.2)	102 (68)	—	<0.01
Dyspnoea	414 (47)	(0.1)	80 (47)	(0.6)	250 (44)	—	84 (56)	—	0.04
Unilateral chest pain	335 (38)	(0.1)	79 (46)	(0.6)	185 (33)	—	71 (47)	—	<0.01
Runny nose	261 (29)	(0.1)	54 (31)	—	153 (27)	(0.2)	54 (36)	—	0.10
Wheezing perceived by patients	187 (21)	(0.1)	47 (27)	(0.6)	95 (17)	—	45 (30)	—	<0.01
Maximal declared temperature (°C)	39 [1]	(14.1)	39 [1]	(17.4)	39 [1]	(12.1)	39 [1]	(17.9)	0.04
Temperature (measured or reported) >37.8 °C	700 (93)	(15.1)	132 (95)	(19.2)	454 (92)	(12.8)	114 (94)	(19.2)	0.60
Tachycardia (⩾100 beats/min)	126 (15)	(5.8)	32 (20)	(7.0)	74 (14)	(5.5)	20 (14)	(5.3)	0.15
Unilateral crackles	684 (77)	—	172 (100)	—	361 (64)	—	151 (100)	—	<0,01
Bilateral crackles	27 (3)	—	0 (0)	—	25 (4)	—	0 (0)	—	—
Unilateral decreased vesicular breathing	222 (25)	—	44 (29)	—	131 (23)	—	47 (27)	—	0.25
Wheezes	166 (19)	—	43 (25)	—	89 (16)	—	34 (23)	—	0.01
Rhonchi	289 (33)	—	62 (36)	—	185 (33)	—	42 (28)	—	0.28
Gennis’ score^d^=0 (no vital sign)	67 (8)	(8.5)	12 (8)	(10.5)	41 (8)	(8.0)	14 (10)	(7.9)	0.69
CRB-65 score^e^=0	399 (62)	(27.4)	65 (55)	(31.4)	269 (66)	(29.0)	65 (57)	(24.5)	0.08
=1	205 (32)		41 (35)		120 (29)		44 (39)		
=2	32 (5)		9 (8)		18 (4)		5 (4)		
=3	6 (1)		3 (3)		3 (1)		0 (0)		
C-reactive protein <20 mg/l	74 (23)	(63.8)	3 (11)	(84.3)	53 (22)	(57.2)	18 (34)	(64.9)	0.16
20–100 mg/l	118 (37)		12 (44)		87 (36)		19 (36)		
>100 mg/l	129 (40)		12 (44)		101 (42)		16 (30)		
White blood cells count >10,000/mm^3^	143 (44)	(63.5)	13 (50)	(84.9)	104 (43)	(57.2)	26 (46)	(62.9)	0.75

Univariate analysis in the population with chest radiography.

Abbreviations: BMI, body mass index; CAP, community-acquired pneumonia; PPV, pneumococcal polysaccharide vaccine.

a Percentages are calculated on available data.

b Data not available.

c Living in a city of <5,000 inhabitants (inhab).

d Score^18^: 1 point for each feature: confusion, respiratory rate ⩾30/min, systolic blood pressure <90 mm Hg or diastolic blood pressure ⩽60 mm Hg, age ⩾65 years.

e Score^17^: 1 point for each feature:temperature >37.8 °C, pulse >100 beats/min, respiratory rate >20/min.

**Table 3:** General practitioner (GP) decisions at the inclusion visit and evolution of patients with positive or negative radiography and without chest radiography

	*All included patients,* N*=886*		*No chest radiography,* N*=172*		*Positive chest radiography,* N*=563*		*Negative chest radiography*, N*=151*	
	n *(%)* a *or mean [s.d.]*	*NA* b *(%)*	n *(%)* a *or mean [s.d.]*	*NA* b *(%)*	n *(%)* a *or mean [s.d.]*	*NA* b *(%)*	n *(%)* a *or mean [s.d.]*	*NA* b *(%)*
First-line antibiotherapy at inclusion	836 (95)	—	166 (96)	—	525 (93)	—	145 (96)	—
Prescription of a sick leave	307 (35)	—	51 (30)	—	213 (38)	—	43 (28)	—
Worsening at the first follow-up visit	49 (10)	(0.6)	8 (10)	(1.2)	36 (11)	(0.6)	5 (5)	—
Duration of fever >38.5 °C (days)	5 [3]	—	4 [2]	—	5 [3]	—	4 [2]	—
Fever duration after the initiation of antibiotics	3 [2]	—	3 [1]	—	3 [2]	—	3 [1]	—
Duration of cough (days)	14 [10]	(7.2)	12 [9]	(22.1)	15 [11]	(2.5)	13 [8]	(7.5)
Duration of sick leave (days)	6 [3]	—	6 [3]	—	7 [3]	—	6 [3]	—
Weakness impacting daily activities (days)	11 [9]	(16.4)	9 [6]	(15.1)	12 [9]	(13.4)	11 [12]	(29.5)
Hospitalisation	62 (7)	(3.4)	11 (6)	(16.3)	45 (8)	(0.2)	6 (4)	(0.7)
Death	3 (0)	(4.2)	1 (1)	(18.6)	1 (0)	(0.9)	1 (1)	—

a Percentages are calculated on available data.

b Data not available.

## Notes

HP, CBdV, VP, AL, CG and SG report having received grant support through the Collège National des Généralistes Enseignants (CNGE) from Pfizer. The remaining authors declare no conflict of interest.
